# Identification of microRNAs implicated in modulating resveratrol-induced apoptosis in porcine granulosa cells

**DOI:** 10.3389/fcell.2023.1169745

**Published:** 2023-05-05

**Authors:** Huibin Zhang, Jinglin Wang, Fan Xie, Yangguang Liu, Mengyao Qiu, Zheng Han, Yueyun Ding, Xianrui Zheng, Zongjun Yin, Xiaodong Zhang

**Affiliations:** ^1^ College of Animal Science and Technology, Anhui Agricultural University, Hefei, China; ^2^ Anhui Province Key Laboratory of Local Livestock and Poultry, Genetical Resource Conservation and Breeding, Hefei, China

**Keywords:** miRNA, resveratrol, apoptosis, ovary, pig

## Abstract

MicroRNAs (miRNAs) are small, noncoding RNAs that play a crucial role in the complex and dynamic network that regulates the apoptosis of porcine ovarian granulosa cells (POGCs). Resveratrol (RSV) is a nonflavonoid polyphenol compound that is involved in follicular development and ovulation. In previous study, we established a model of RSV treatment of POGCs, confirming the regulatory effect of RSV in POGCs. To investigate the miRNA-level effects of RSV on POGCs to reveal differentially expressed miRNAs, a control group (n = 3, 0 μM RSV group), a low RSV group (n = 3, 50 μM RSV group), and a high RSV group (n = 3, 100 μM RSV group) were created for small RNA-seq. In total, 113 differentially expressed miRNAs (DE-miRNAs) were identified, and a RT-qPCR analysis showed a correlation with the sequencing data. Functional annotation analysis revealed that DE-miRNAs in the LOW vs. CON group may be involved in cell development, proliferation, and apoptosis. In the HIGH vs. CON group, RSV functions were associated with metabolic processes and responses to stimuli, while the pathways were related to PI3K24, Akt, Wnt, and apoptosis. In addition, we constructed miRNA-mRNA networks related to Apoptosis and Metabolism. Then, ssc-miR-34a and ssc-miR-143-5p were selected as key miRNAs. In conclusion, this study provided an improved understanding of effects of RSV on POGCs apoptosis through the miRNA modulations. The results suggest that RSV may promote POGCs apoptosis by stimulating the miRNA expressions and provided a better understanding of the role of miRNAs combined with RSV in ovarian granulosa cell development in pigs.

## 1 Introduction

The ovaries—as gonads producing oocytes and as endocrine glands producing hormones that provide an environment suitable for fertilization, implantation and pregnancy—are extremely important for female reproduction (Zhu et al., 2019). Only less than 1% of follicles mature and ovulate, and the vast majority of mammalian follicles undergoes atresia (Liu et al., 2014). Proliferation and apoptosis of ovarian granulosa cells play key roles in follicular development and atresia, which are natural phenomena occurring in healthy female mammals, both prenatally and postnatally. Therefore, it is important to elucidate the molecular mechanisms and regulatory networks of follicular atresia and granulosa cell apoptosis to maintain normal development of mammalian ovarian functions.

Resveratrol (3,4,5-trihydroxystilbene, RSV), a polyphenol and well-known natural antioxidant, has been detected in grapes, peanuts, and in more than 70 species of plants (Kong et al., 2011; Sarkar and Pal, 2014; Jin et al., 2016). Previous findings have indicated that RSV exhibits antioxidant, anti-inflammatory, and growth-inhibiting activities in several cancer cell lines and primary cells (Manna et al., 2000; Gusman et al., 2001; Joe et al., 2002). These properties have been linked to the inhibition of proliferation-related cell cycle arrest and apoptotic cell death, typically observed *in vitro* at concentrations ranging from 25 to 400 µM (Wong et al., 2010). Moreover, RSV interferes with cell cycle progression through blocking the G1/S or G2/M phase of different cancer cells (Larrosa et al., 2003; Lee et al., 2004; Gatouillat et al., 2010) and also regulates cell cycle arrest and apoptosis through the p27^KIP1^ and p53/p21^WAF1/CIP1^ pathways (Singh et al., 2017). It has been confirmed that the RSV 2-hydroxy analog has biological activity in porcine ovarian granulosa cells. It inhibited cell viability and progesterone and estradiol production in a dose-dependent manner in porcine ovarian granulosa cells (Basini et al., 2010). In short, RSV has a number of properties which enable its influence on female reproduction at various regulatory levels via various extra- and intracellular signaling pathways (Sirotkin, 2021).

MiRNAs are short, noncoding regulatory RNAs that play key roles in the restoration of cellular homeostasis or adaptation to environmental conditions through controlling mRNA translation and stability (Aires et al., 2017). miRNAs participate in important biological processes, such as cell proliferation, differentiation, apoptosis, and metabolism *in vivo* through regulating the expression of target genes (Brennecke et al., 2003; Bartel, 2004; Chen et al., 2004; Poy et al., 2004). It has been reported that nuclear-enriched miR-195-5p may be associated with porcine ovarian follicular development and maturation (Bai et al., 2021), and miR-181a regulates porcine granulosa cells apoptosis by targeting TGFBR1 via the activin signaling pathway (Zhang et al., 2020). Notably, some scholars have systematically reported the role of miRNAs in the porcine ovary, pointing out that let-7 family, miR-23-27-24 cluster, miR-183-96-182 cluster and miR-17-92 cluster are associated with porcine follicular atresia (Zhang J. et al., 2019), and miR-125b is a potent physiological inhibitor of porcine granulosa ovarian cell functions-cell cycle, apoptosis, and secretory activity (Fabová et al., 2023). In addition, miR-1343 promotes porcine granulosa cell proliferation and inhibits apoptosis (Hu et al., 2021). Therefore, the potential functions of miRNAs in porcine ovarian granulosa cells are worth investigating.

Our previous study focused on lncRNA-mRNA changes in porcine ovarian granulosa cells (POGCs) after RSV treatment and was conducted to identify the differentially expressed lncRNAs and mRNAs (Zhang et al., 2023); however, it is unknown whether miRNAs changed in POGCs after RSV treatment. Therefore, this study mainly focuses on the identification of the specific miRNAs influenced by RSV treatment on POGCs apoptosis, which aims to investigate the roles of RSV in including miRNA for the better understanding, and to reveal new candidate regulatory miRNAs and genes underlying the apoptosis of POGCs. The miRNA expression profiles in the three groups of POGCs treated with different concentrations of RSV were explored in this study. Gene Ontology (GO) enrichment and Kyoto Encyclopedia of Genes and Genomes (KEGG) pathway analyses were performed to reveal a number of biological processes driven by RSV treatment. Some potentially functional miRNA-mRNA networks have also been identified. These screened data provided references for the apoptosis of POGCs and application of RSV in clinical medicine and animal husbandry production.

## 2 Materials and methods

### 2.1 Ethics approval and consent to participate

All experiments were performed in accordance with the relevant guidelines and regulations and adhered to the ARRIVE guidelines for reporting animal experiments. This study was conducted according to the principles of the Basel Declaration and recommendations of the Guide for the Care and Use of Laboratory Animals. The protocol was approved by the Ethics Committee of the Anhui Agricultural University under permit no. AHAU20201025.

### 2.2 Culture POGCs treated with RSV

Fresh pig ovaries were collected from Landrace gilts (approximately 1 year old, average weight about 150 kg, in the follicular phase) at a local slaughterhouse (Hefei, Anhui, China).

Brightly colored ovaries with a large number of antral follicles and fullness were selected. Small, healthy follicles with pink and well vascularized walls between 3 and 5 mm in diameter were punctured with a disposable syringe, and the follicular fluid was aspirated without disrupting the vessels; Transparent follicular fluid was collected from more than 50 ovaries of gilts and combined to remove the effect of an individual animal. The fluid was centrifuged (1,200 × g, 5 min), resuspended and centrifuged again. Then, the POGCs (pellet) were immediately seeded in 6-well plates and cultured (10^6^ viable cells/well) in a humidified atmosphere (5% CO_2_, 95% air, 37°C) in Dulbecco’s modified Eagle’s medium supplemented (Invitrogen, Carlsbad, CA, United States) with 10% fetal bovine serum and 1% penicillin/streptomycin mixture (Invitrogen). When the POGCs reached 80% confluency, the medium was removed, and the cells were treated with 0, 50 and 100 μM of RSV (Solarbio, Beijing, China; Zhang et al., 2023), representing control, LOW and HIGH group, respectively. The POGCs were cultured with treatments in triplicate for 24 h under conditions described above. At the end of the culture, the cells were harvested and used for RNA isolation.

### 2.3 RNA extraction and small RNA library construction

Total RNA was extracted from POGC samples using the TRIzol reagent (Invitrogen) according to the manufacturer’s protocol. Possible degradation and contamination of the RNA samples were evaluated via 1% agarose gel electrophoresis. RNA quality was detected using the NanoPhotometer^®^ spectrometer (IMPLEN, CA, United States) and Agilent Bioanalyzer 2,100 system (Agilent Technologies, CA, United States). RNA extracts with OD_260/280_ absorbance between 1.8 and 2.0 and RNA integrity number equal to or higher than 7.0, were used for further experiments. The *Sus scrofa* genome (Sscrofa v11.1) was selected as the reference genome for this study.

Next, small RNA (sRNA) -seq was performed to identify the differentially expressed miRNAs (DE-miRNAs) in POGCs. The NEBNext^®^Multiplex Small RNA Samples Prep Kit Set was used with approximately 3 μg of total RNA per sample for the construction of sRNA libraries on the Illumina^®^ (NEB, MA, United States). Briefly, 3′SR and 5′SR adaptors for Illumina were ligated to the sRNA. Reverse transcription of the synthetic first chain and PCR amplification were performed using LongAmp Taq 2X Master Mix, SR Primer for Illumina and index (X) primer. The PCR products were purified on 8% polyacrylamide gel (100 V, 80 min), and library quality was assessed using the Agilent Bioanalyzer 2,100 system. Finally, clustering of the indexed samples was performed on a cBot Cluster Generation System using TruSeq SR Cluster Kit v3-cBot-HS (Illumina) according to the manufacturer’s instructions; 50-nt single-end reads were generated.

### 2.4 Identification of miRNA

The raw reads of the sRNA-seq were first processed using custom Perl and Python scripts. In this step, clean reads were obtained through removing reads containing poly-N (with 5′adapter contaminants and poly-A, -T, -G, or -C, without 3′adaptor or the insert tag) and low-quality reads from the raw data. The Q20, Q30, and GC content of the clean data were calculated for all RNAs, and 18–35 nt fragments from the clean reads were selected for downstream analysis. The raw miRNA data were stored in FASTQ format. To ensure the accuracy of our subsequent analysis, all clean reads were aligned to the *Sus scrofa* genome using the Bowtie software (v1.1.2) (Langmead et al., 2009). Mapped sRNA tags were aligned with the specified range sequencing in miRBase20.0 for miRNA identification, mirdeep2 and srna-tools-cli were used to obtain potential miRNAs and draw secondary structures. Based on the custom script, the number of miRNAs was obtained, as well as the base bias at the first location of a certain length of miRNAs and at each location of all identified miRNAs. Clean reads that were not annotated as known miRNAs were compared with the RepeatMasker and Rfam database from the specified species, and tags originating from protein-coding genes, repeat sequences, rRNA, tRNA, snRNA, and snoRNA were removed. Finally, the novel miRNAs were identified using the miRNA prediction software miREvo and mirdeep2 through exploring the secondary structure.

### 2.5 Expression, target genes, and function analysis of miRNAs

The expression of known and novel miRNAs in each sample was quantified, and the transcript per million (TPM) values were used to assess miRNA expression. The target genes of miRNAs were then analyzed using miRanda. Subsequently, differential expression analysis of POGCs was performed using the DESeq R package (3.0.3), and DE-miRNAs were identified based on the following criteria: log_2_|Fold Change| > 0.585 and *p*-value < 0.05.

To explore the functional category distribution and pathway enrichment of the miRNA targets, we performed GO enrichment and KEGG pathway analyses of the predicted target genes of DE-miRNAs. All GO categories and KEGG pathways were screened with a *p*-value < 0.05.

### 2.6 Real-time polymerase chain reaction

The CFX96 Real-Time System (BioRad, CA, United States) was used to validate the expression levels of DE-miRNAs. cDNA was synthesized using the Mir-^X^™ miRNA First-Strand Synthesis Kit (TaKaRa, Dalian, China). The primer sequences for the selected miRNAs are listed in Supplementary Table S8. Quantitative real-time PCR (qRT-PCR) was performed in a 20 µL reaction mixture containing 10 µL 2 × iTaqTM Universal SYBR^@^ Green Supermix (BioRad, CA, United States), 1 µL cDNA, 8 µL ddH2O, and 0.5 µL each forward and reverse primers. The *U6* housekeeping gene was used to normalize the expression levels of the miRNAs, and the relative expression levels were calculated via 2^−ΔΔCT^ method. Repeat the experiment at triplicates for each sample to reduce experimental error.

### 2.7 Statistical data analysis

**FIGURE 1:**
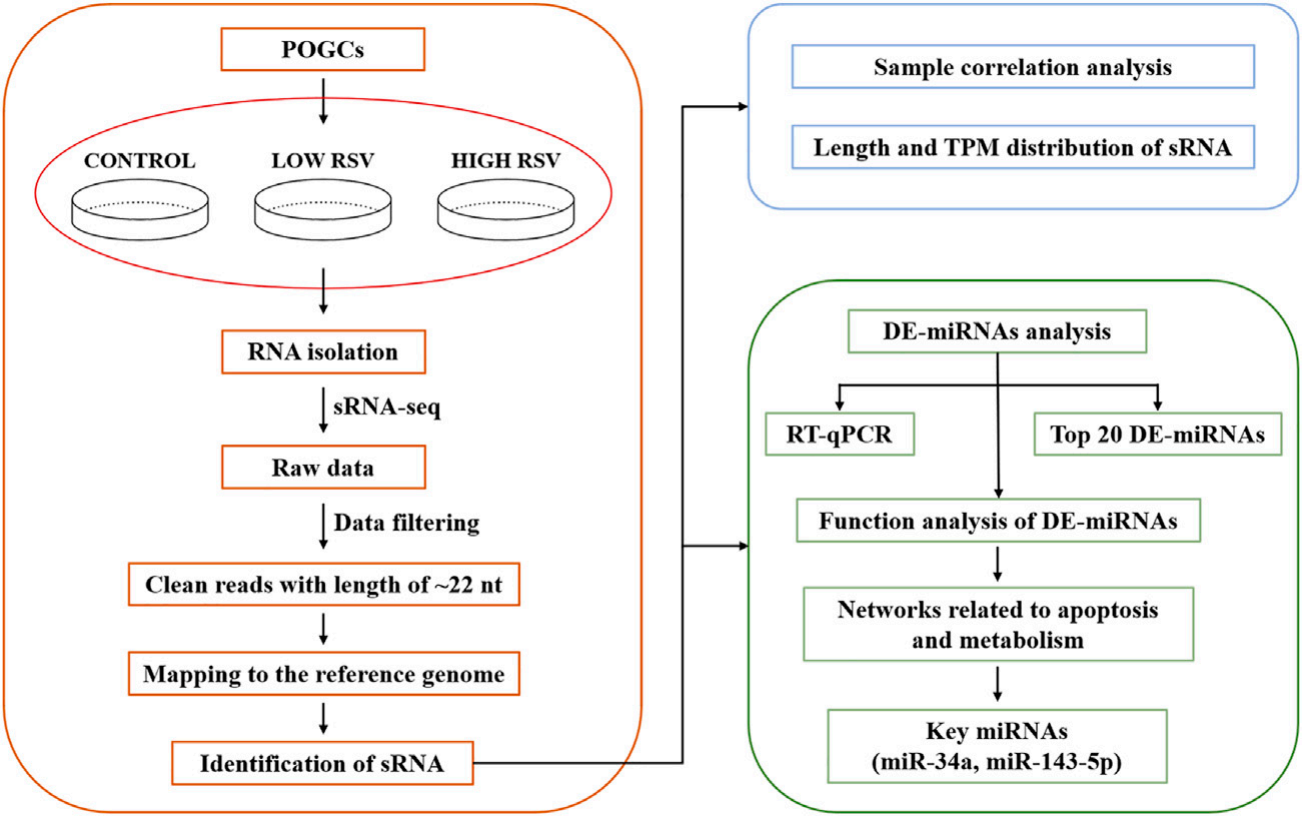
The method pipeline and experimental design used to identify miRNAs involved in porcine ovarian granulosa cells treated with resveratrol.

The RT-qPCR data are presented as means ± standard deviation (SD) for at triplicates. The GraphPad Prism (version 5.0) software (San, Diego, CA, United States) was used to analyze the results of RT-qPCR and for graphing. One-way ANOVA of the normalized data was then conducted using GraphPad Prism for Windows. Duncan’s multiple comparisons was performed to test for significant differences between the mean values at different stages. For the above experiments, we carried out a workflow flow chart (Figure 1).

## 3 Results

### 3.1 Overviews of POGCs small RNA-seq

**FIGURE 2:**
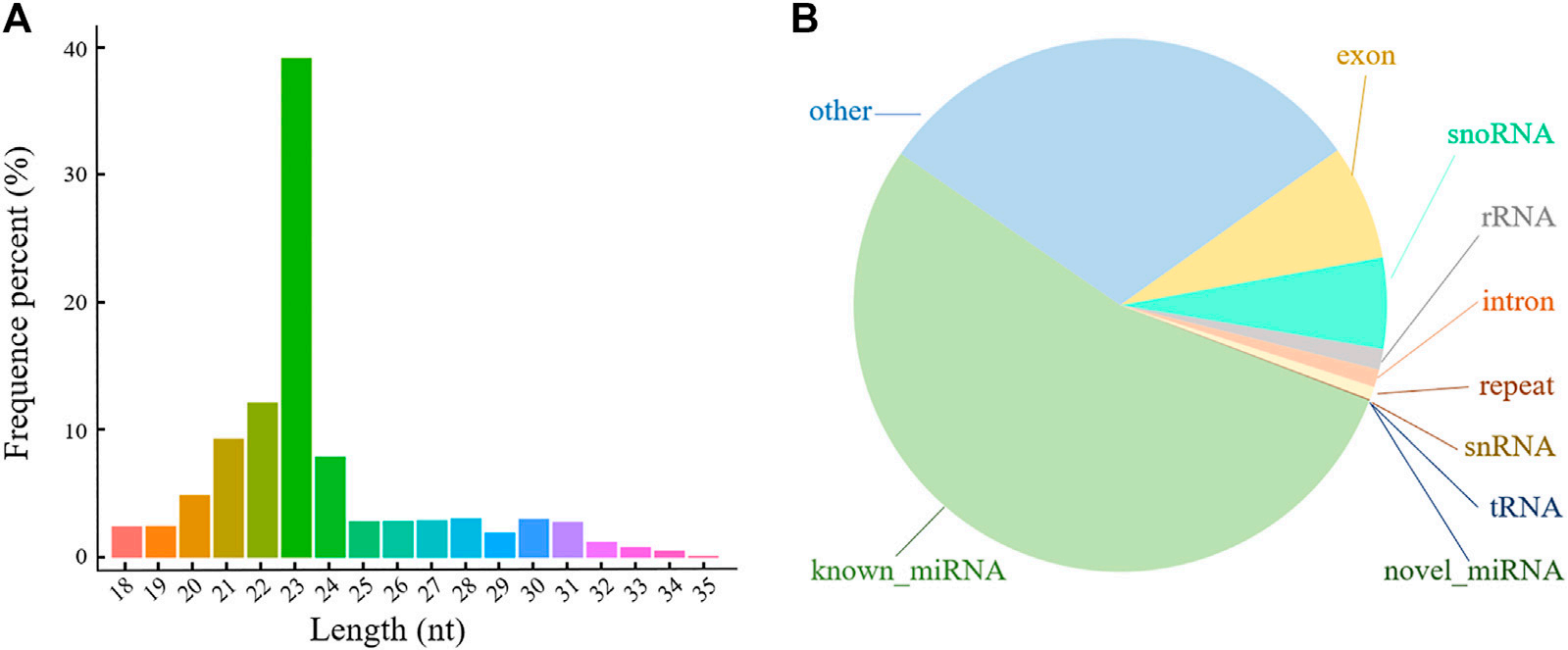
Length and classification of all sRNAs in porcine ovarian granulosa cells treated with resveratrol. **(A)** Length distribution of all sRNAs. **(B)** Classification pie chart of all sRNAs. Different colors represent the proportion of different types of sRNA.

To determine the role of miRNAs in POGC apoptosis, nine miRNA-seq libraries were used to screen the POGC miRNAs: blank group (control group, CON), low-concentration treatment group (50 μM RSV, LOW), and high-concentration treatment group (100 μM RSV, HIGH), each with three biological replicates. A total of 12.43–16.64 million paired raw reads were generated by the Illumina Hiseq™ 2,500 platform for each library with a Q30 of 92.10%–97.56% (Supplementary Table S1). After the libraries were filtered, 11.22–15.66 million single-end reads (approximately 94.43%) acquired from the three groups (CON, LOW, and HIGH) were classified as miRNAs. After length screening, 7.66–12.94 million clean reads were considered as sRNA reads, of which 92.33%–98.68% were mapped to the genome (Supplementary Table S1). The mapped sRNA reads were used for known and novel miRNA identification and sRNA annotation. The results showed that the lengths of sRNA range from 18 to 35 nt (Figure 2A), most of which were 20–24 nt-long, indicating a normal distribution of sRNA lengths compared with other sRNA-seq studies. Furthermore, all known and novel miRNAs accounted for the largest proportion of sRNAs at about 53.8%, and rRNA accounted for less than 1.25% (Figure 2B), indicating that the data were reliable.

**FIGURE 3:**
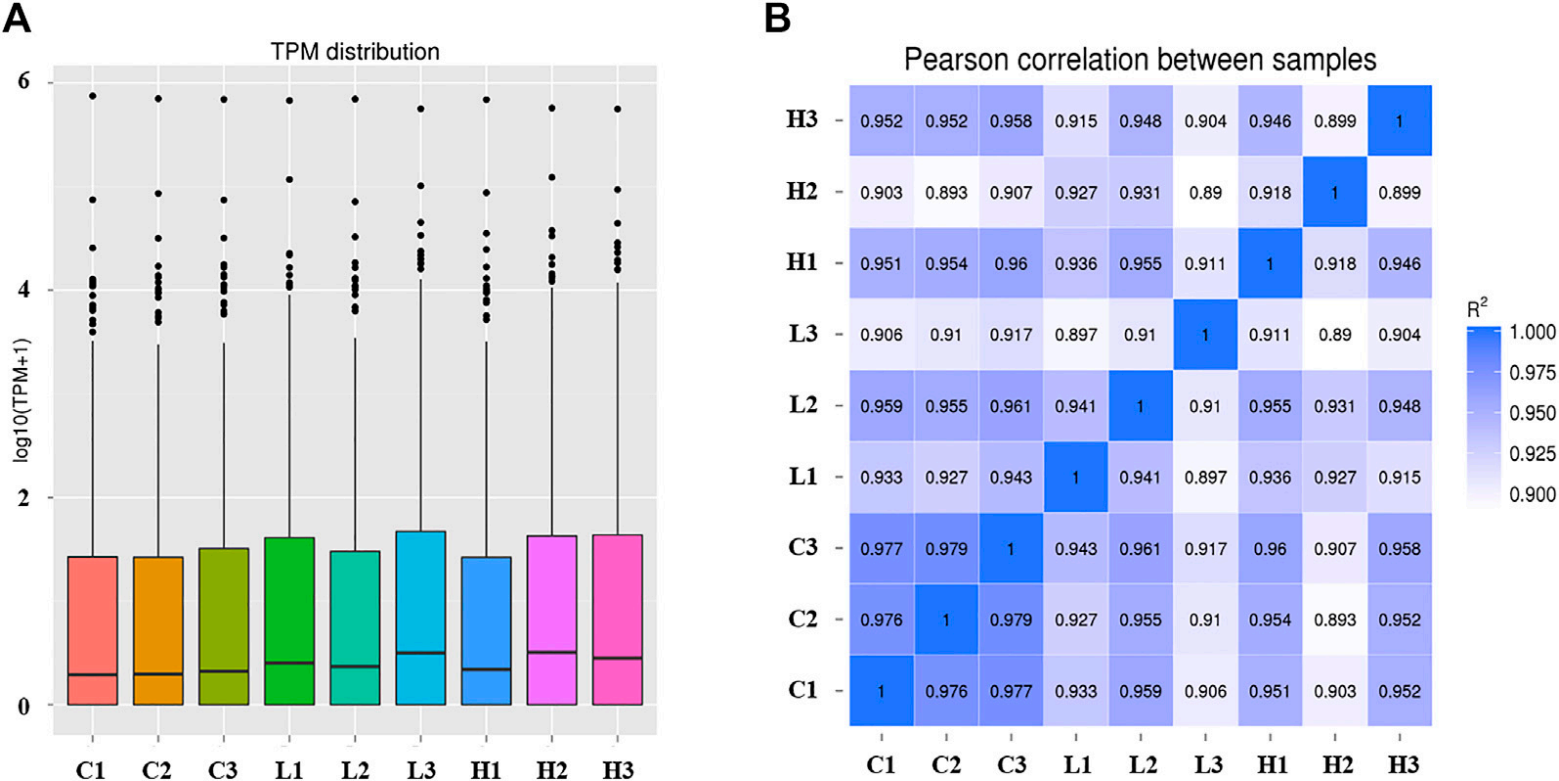
Expression level and correlation analysis of samples. **(A)** Transcripts per million (TPM) distribution of miRNAs. The abscissa is the sample name. The ordinate is log_10_ (TPM+1). The box plot of each area corresponds to five statistics (top to bottom: maximum value, upper quartile, median value, lower quartile, and minimum value). **(B)** Sample correlation analysis of all miRNAs. The color spectrum, ranging from white to blue, represents Pearson correlation coefficients from 1 to 0, indicating high to low correlations.

The TPM distribution of the miRNAs was explored (Figure 3A; Supplementary Figure S1). The distribution of the known miRNAs in each group was balanced (Figure 3A), and sample correlation analysis showed that the three replicates of each group had the highest correlation (Figure 3B). Ultimately, 546 miRNAs were identified in the POGCs of CON, LOW, and HIGH groups, which included 332 known and 214 novel miRNAs (Supplementary Table S2).

### 3.2 Differential expression analysis of miRNA in POGCs

**FIGURE 4:**
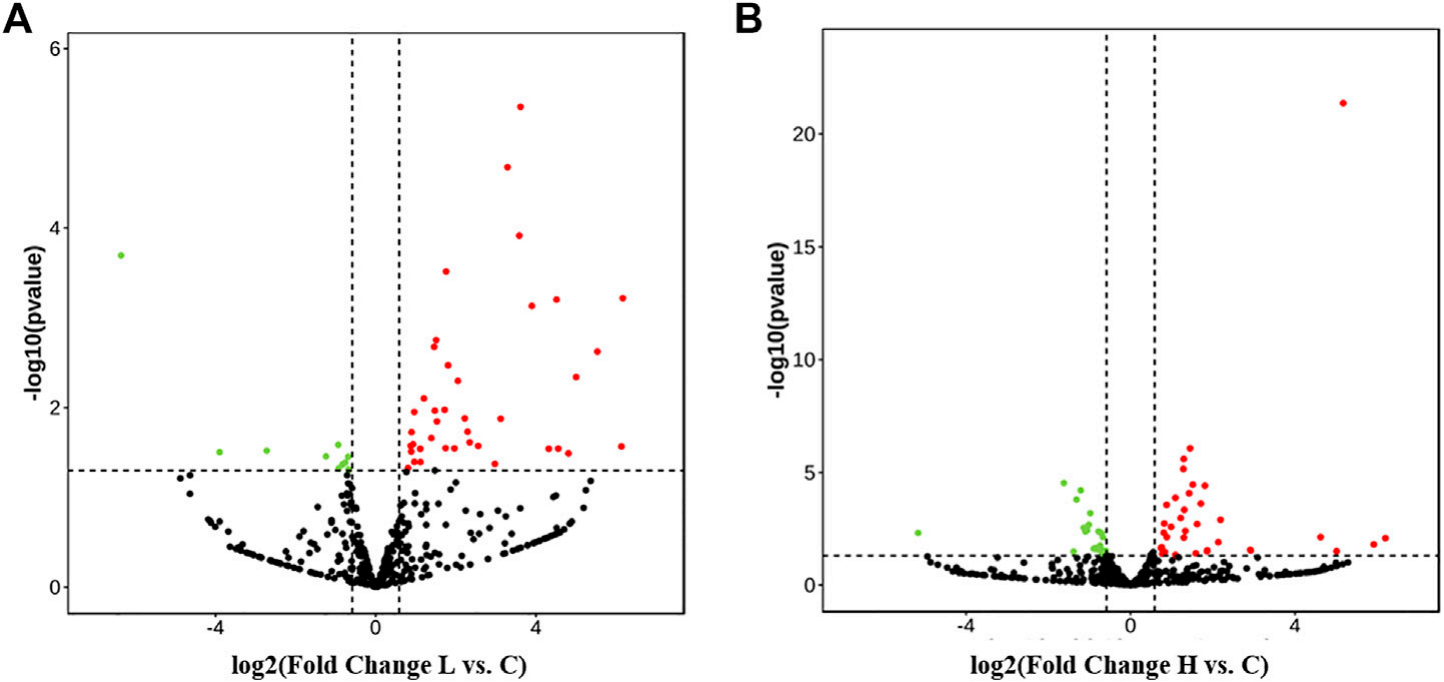
Expression Profiles of miRNAs in porcine ovarian granulosa cells treated with resveratrol. **(A)** Volcano plots of differentially expressed miRNAs (DE-miRNAs) in the POGCs of the LOW vs. CON group. **(B)** Volcano plot of DE-miRNAs in the POGCs of the HIGH vs. CON group. Red and green show upregulated and downregulated miRNAs, respectively.

Considering that there were three different treatment concentrations and two comparisons, we performed the analysis for the two comparisons. The DE-miRNAs in the samples are shown using volcano plots (Figure 4), and a heatmap (Supplementary Figure S2). The results showed that there were 52 DE-miRNAs (41 upregulated and 11 downregulated) in the LOW vs. CON group (Figure 4A; Supplementary Table S3). In addition, the results of the HIGH vs. CON comparison were different from the previous ones, and we found 61 DE-miRNAs, among which 36 were upregulated and 25 DE-miRNAs were downregulated (Figure 4B, Supplementary Table S3). As shown in Table 1, for the LOW vs. CON group, the most upregulated and downregulated miRNAs were ssc-miR-10390 (6.17-fold change) and novel_297 (6.36-fold change), respectively. For the HIGH vs. CON group, the most upregulated and downregulated miRNAs were novel_372 (6.20-fold change) and ssc-miR-190a (5.20-fold change), respectively. The top ten upregulated and downregulated miRNAs are listed in Table 1. To further understand the role of DE-miRNAs in POGCs treated with RSV, the target genes of DE-miRNAs were selected by GO enrichment and KEGG pathway analyses.

**TABLE 1:** Top20 differentially expressed miRNA statistics.

A) The ten most significantly up- and downregulated miRNAs in HIGH vs. CON.				
sRNA	log2FoldChange	*p*-value	p-adj	Regulation
ssc-miR-190a	−5.17	0.0048666	0.071154	down
ssc-miR-374a-5p	−1.63	2.95E-05	2.40E-03	down
ssc-miR-503	−1.39	3.26E-02	2.67E-01	down
ssc-miR-500-5p	−1.32	1.62E-04	0.0058385	down
ssc-miR-374b-5p	−1.21	6.24E-05	0.0033796	down
ssc-miR-17-5p	−1.14	2.86E-03	0.05388	down
ssc-miR-107	−1.10	0.0041929	0.067242	down
ssc-miR-7857-3p	−1.06	0.003744	0.067242	down
ssc-miR-7-5p	−1.02	0.0021056	0.043416	down
ssc-miR-16	−0.98	6.42E-04	0.017371	down
ssc-miR-219b-3p	1.86	2.89E-02	0.26368	up
ssc-miR-126-5p	2.13	1.25E-02	1.46E-01	up
ssc-miR-200b	2.19	1.27E-03	0.030532	up
ssc-miR-206	2.91	2.73E-02	0.25679	up
novel_306	2.92	2.92E-02	2.64E-01	up
novel_635	4.62	7.40E-03	9.56E-02	up
ssc-miR-10a-3p	5.01	3.14E-02	0.26522	up
ssc-miR-202-3p	5.17	4.2483E-22	1.8395E-19	up
novel_394	5.91	1.57E-02	0.17923	up
novel_372	6.20	0.008292	0.099734	up

B) The ten most significantly up- and downregulated miRNAs in LOW vs. CON.				
sRNA	log2FoldChange	*p*-value	p-adj	Regulation
novel_297	−6.36	0.00020156	0.015168	down
novel_365	−3.90	3.14E-02	2.36E-01	down
ssc-miR-454	−2.72	3.03E-02	2.36E-01	down
ssc-miR-450a	−1.24	3.50E-02	0.24718	down
ssc-miR-378b-3p	−0.93	2.60E-02	0.23436	down
ssc-miR-7857-3p	−0.93	4.78E-02	0.28426	down
ssc-miR-450c-5p	−0.83	0.042994	0.26961	down
ssc-miR-374b-5p	−0.76	0.041011	0.26835	down
ssc-miR-378	−0.69	0.035312	0.24718	down
ssc-miR-103	−0.68	4.87E-02	0.28426	down
ssc-miR-10390	6.17	6.03E-04	0.026913	up
novel_710	6.13	2.72E-02	2.34E-01	up
ssc-miR-142-3p	5.54	2.38E-03	0.065103	up
ssc-miR-122-5p	5.01	4.57E-03	0.098332	up
ssc-miR-193a-3p	4.81	3.25E-02	2.38E-01	up
novel_466	4.56	2.87E-02	2.34E-01	up
ssc-miR-34c	4.52	6.26E-04	0.026913	up
ssc-miR-142-5p	4.32	0.028791	0.23436	up
ssc-miR-196a	3.90	7.37E-04	0.027739	up
ssc-miR-96-5p	3.62	4.4594E-06	0.0013423	up

### 3.3 qRT-PCR verification of DE-miRNAs in POGCs

**FIGURE 5:**
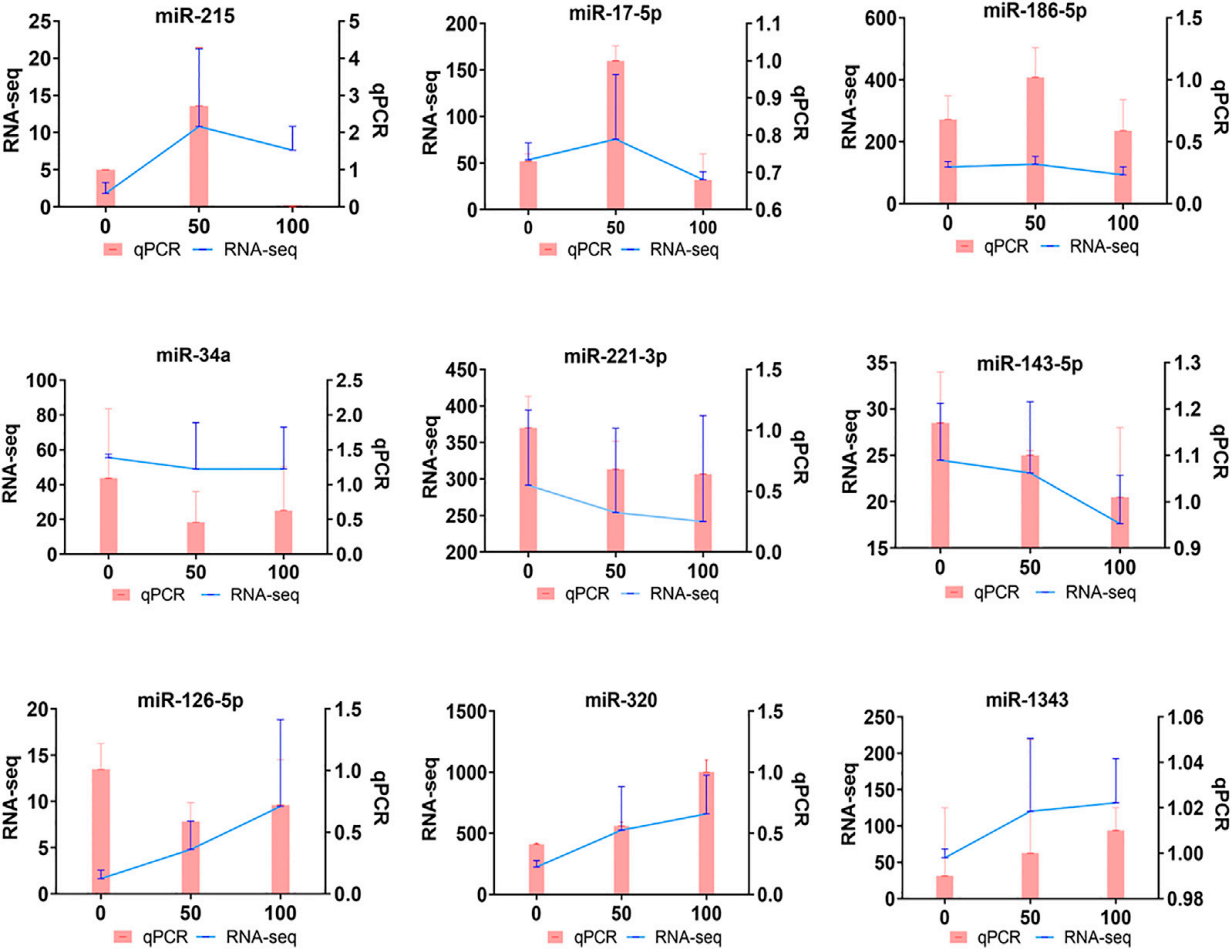
Quantitative real-time PCR (qRT-PCR) verification of DE-miRNAs in porcine ovarian granulosa cells treated with resveratrol. qRT-PCR (Bar chart, pink) and sRNA-seq expression (Line chart, blue) validation of the indicated POGC miRNAs. The miRNA expression levels were normalized to the U6 gene, and sRNA-seq expression was normalized to TPM.

To verify the credibility of sRNA-seq, qRT-PCR was performed on RNA extracted from POGCs to confirm the expression level changes obtained from the sRNA-seq analysis. We selected nine miRNAs to validate the results of sRNA-seq. The miRNAs were verified, and the relative expression levels of validated miRNAs are shown in Figure 5. The qRT-PCR results were consistent with the sRNA-seq results.

### 3.4 Analysis of DE-miRNAs in the LOW vs. CON group

**FIGURE 6:**
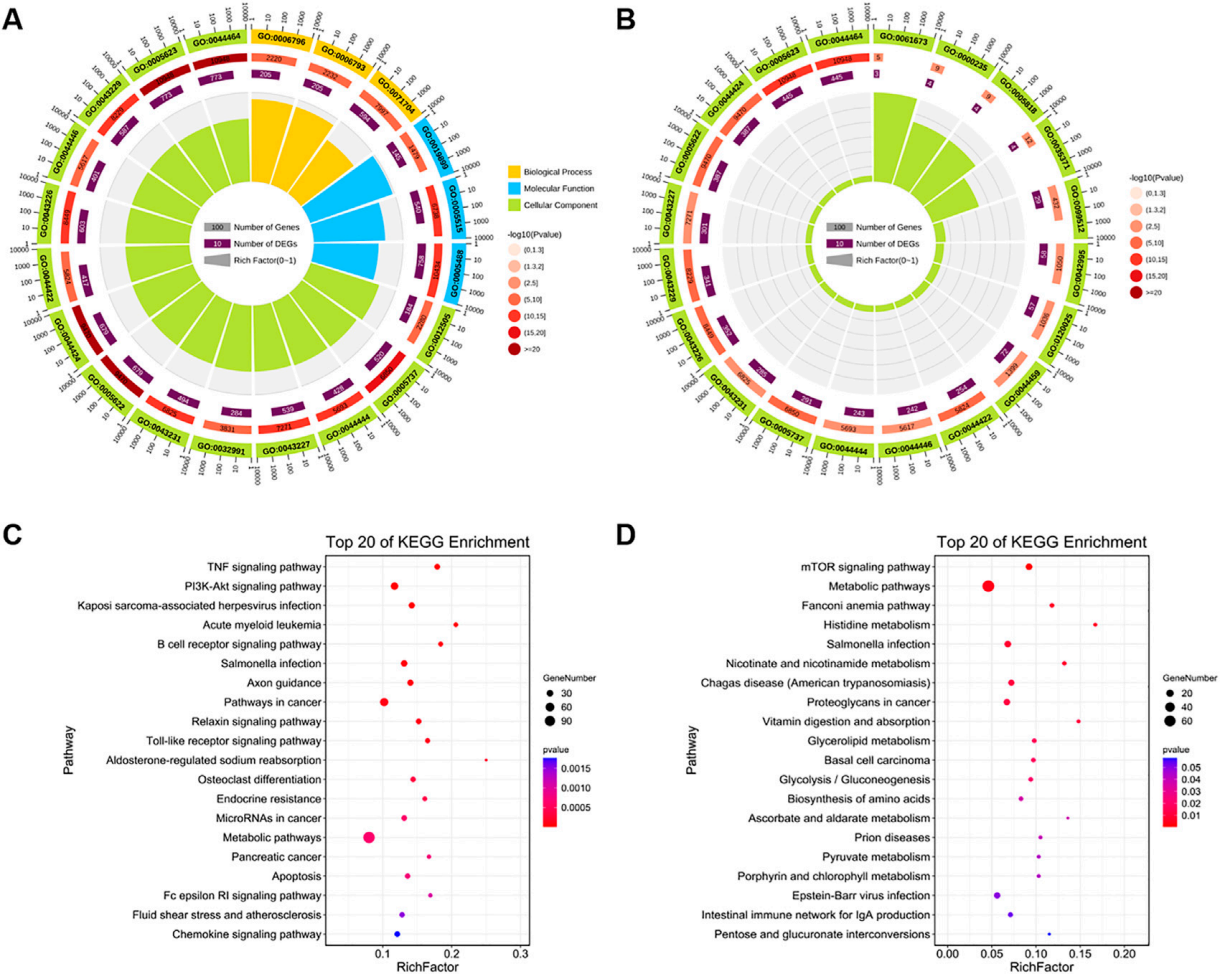
Function and pathway analyses of LOW vs. CON group. **(A,B)** Top 20 gene ontology (GO) enriched terms and **(C,D)** Kyoto Encyclopedia of Genes and Genomes (KEGG) pathways of target genes of upregulated and downregulated DE-miRNAs in the LOW vs. CON group. GO enrichment terms. Different colors represent different categories. The second circle represents the *p*-value and number of background genes in each category. The larger the number of genes, the longer is the bar. The smaller the *p*-value, the darker is the red color. The third circle represents the total number of foreground genes. The fourth circle represents the Rich Factor value of each category (the number of foreground genes in the category divided by the number of background genes). Each grid of the background auxiliary line represents 0.1.

The GO functions and KEGG enrichment pathways of DE-miRNAs that were upregulated and downregulated in the LOW vs. CON group were identified. A total of 1267 target genes were predicted for 41 upregulated DE-miRNAs (Supplementary Table S4), and these genes were enriched in basic functions, such as cytoplasm and plasma and enriched in cell development-related functions, such as protein binding, phosphorylation, metabolic processes, and regulation of molecular function (Figure 6A). Unexpectedly, in the top 20 GO terms, only three terms could be found in the molecular function classification, all of which were related to binding. Moreover, in the classification of biological processes, as in the previous case, there were only three terms, and these were related to metabolism (Supplementary Table S5). For the 11 downregulated DE-miRNAs in the LOW vs. CON group, the 730 target genes corresponded to them (Supplementary Table S4), which were also related to the cytoplasm and plasma functions (Figure 6B). Furthermore, the top 20 GO terms were all associated with cellular components, and most of the GO terms enriched on the target genes of downregulated miRNAs could be found on the same terms on the target genes of upregulated miRNAs (Supplementary Table S5).

For the target genes of the downregulated DE-miRNAs, all KEGG pathways were not significantly enriched (*P*-adj > 0.05), and the data with *p* < 0.05 also showed some interesting results (Supplementary Table S5). Upregulated DE-miRNAs were involved in cell apoptosis and growth, and the enriched pathways were apoptosis, TNF, PI3K-Akt, Toll-like receptor, NOD-like receptor, and sphingolipid signaling pathways (Figure 6C). The target genes of downregulated DE-miRNAs were enriched in the key signaling pathways of cell development, proliferation, and apoptosis (*p* < 0.05), including mTOR, metabolic, vitamin digestion and absorption, biosynthesis of amino acids, and pyruvate metabolism signaling pathways (Figure 6D).

### 3.5 Analysis of DE-miRNAs in the HIGH vs. CON group

**FIGURE 7:**
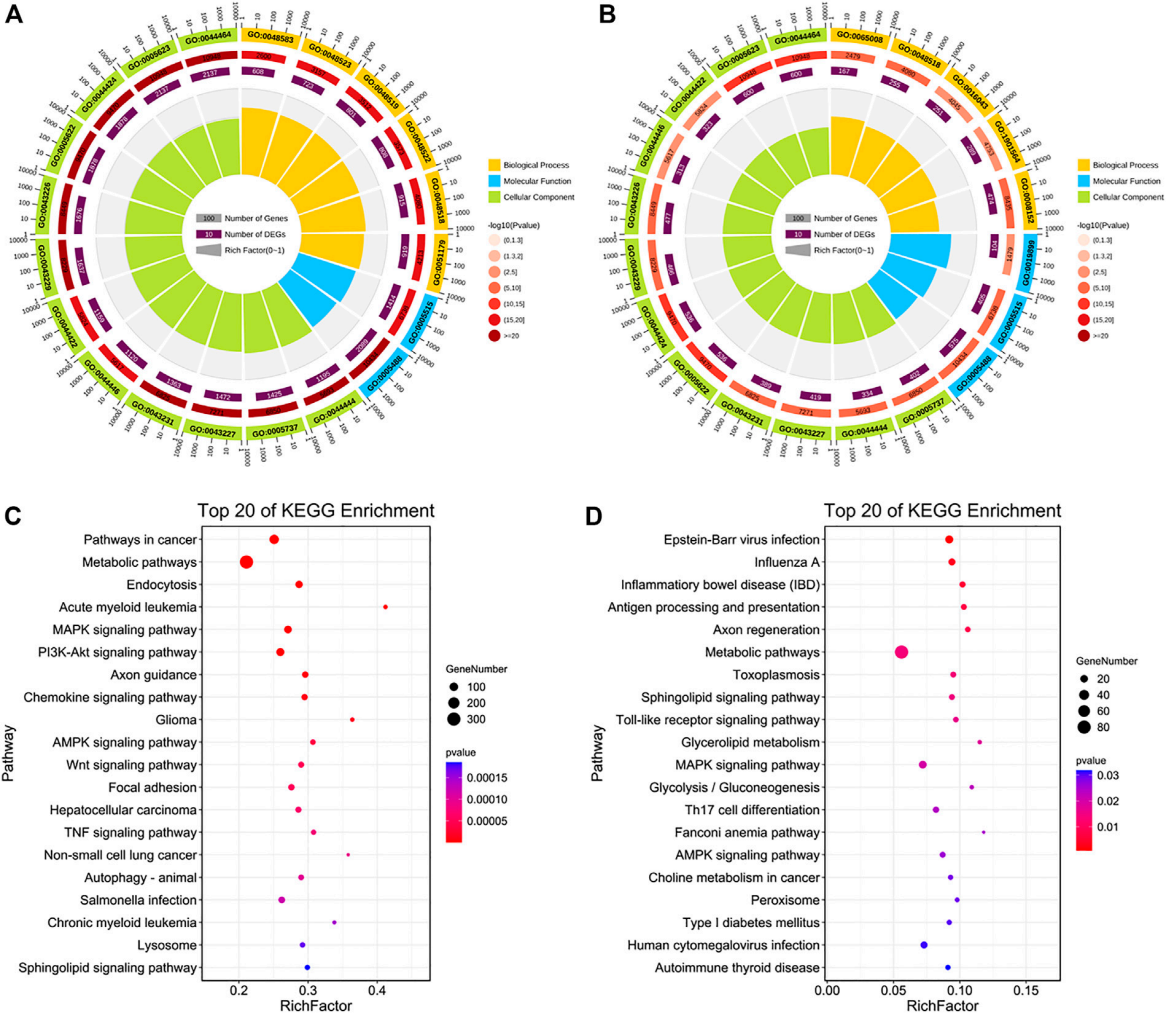
Function and pathway analyses of HIGH vs. CON group. **(A,B)** Top 20 GO enriched terms and **(C,D)** KEGG pathways of target genes of upregulated and downregulated DE-miRNAs in the HIGH vs. CON group.

In the HIGH vs. CON group, the 3,488 target genes of 36 upregulated DE-miRNAs were related to response to stimulus and metabolic process, particularly relevant to some GO terms related to cellular life activities, such as regulation of protein binding, regulation of cell communication, cellular metabolic processes, and regulation of signal transduction (Figure 7A; Supplementary Tables S4, S6). We found that only two GO terms related to molecular function were enriched to the top 20, which are associated with binding. The 951 target genes of 25 downregulated DE-miRNAs enriched GO terms related to metabolic processes and regulation of response to stimulus (Figure 7B). We found three molecular functions related to GO terms, which are related to protein and enzyme binding, that are largely consistent with those described earlier in the LOW vs. CON group.

Next, KEGG pathways with *p* < 0.05 were identified (Supplementary Table S6). Enriched terms of upregulated DE-miRNAs showed that they were involved in metabolic, MAPK, PI3K-Akt, chemokine, AMPK, Wnt, and TNF signaling pathways (Figure 7C). Target genes of downregulated DE-miRNAs participated in the Toll-like receptor, sphingolipid, fatty acid biosynthesis, MAPK, PI3K-Akt, and apoptosis signaling pathways (Figure 7D).

### 3.6 Screening of key miRNAs associated with POGC apoptosis induced by RSV

**FIGURE 8:**
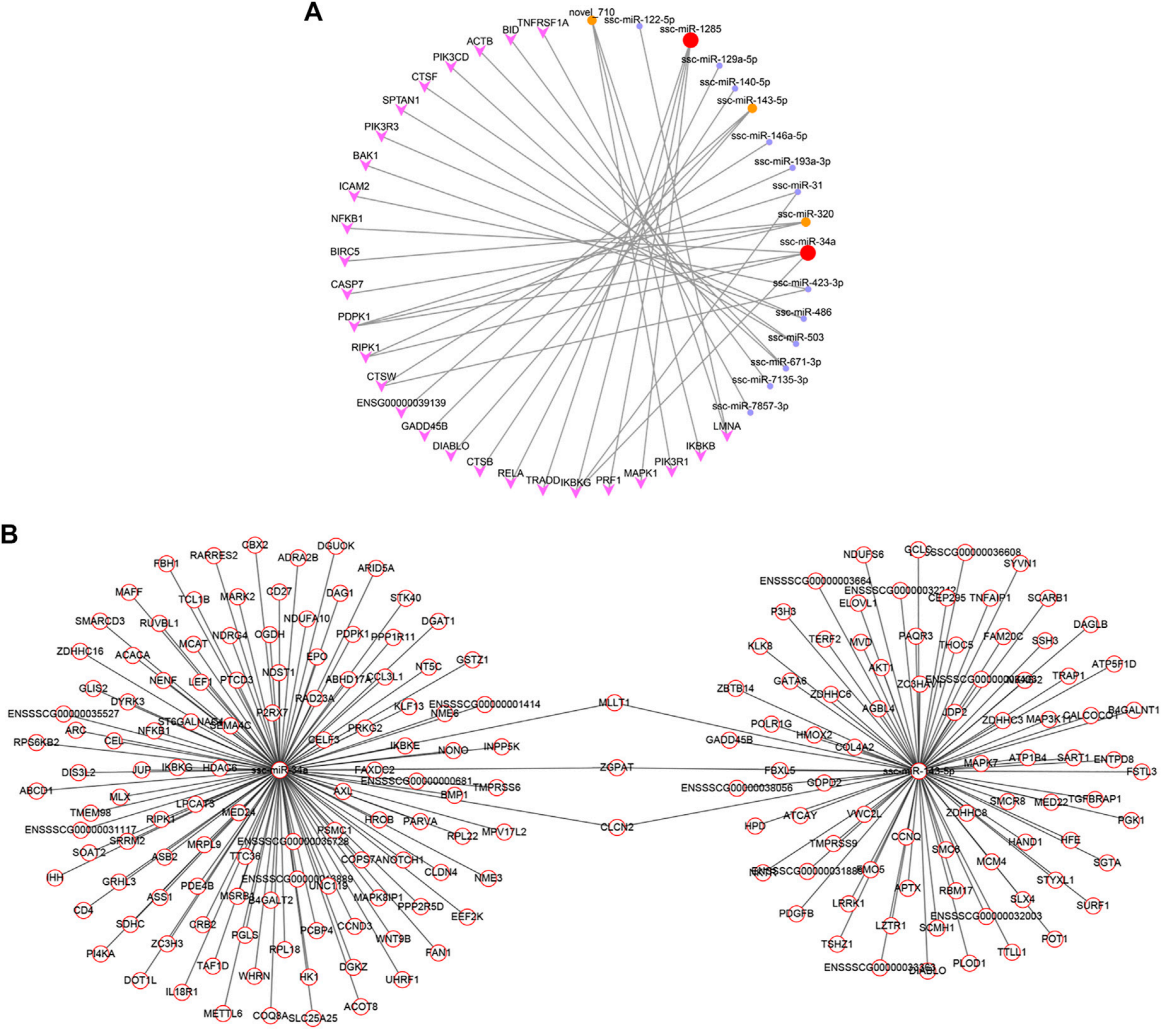
Constructed networks based on the selected vital miRNAs related to resveratrol and porcine ovarian granulosa cells apoptosis. **(A)** miRNA-mRNA network for “Apoptosis”. **(B)** miRNA-mRNA network for “Metabolism”.

The main purpose of this study was to investigate the effects of RSV on POGCs. It was confirmed that apoptosis-related GO terms and pathways were significantly enriched in the GO enrichment and KEGG pathway analyses. Therefore, we screened the key miRNAs involved in apoptosis and a miRNA-mRNA network for the “apoptosis signaling pathway” (Figure 8A; Supplementary Table S7). The 17 miRNAs involved in the pathway targeted 27 genes, of which ssc-miR-34a and ssc-miR-1285 had four target genes. Moreover, ssc-miR-143-5p, ssc-miR-320, and novel_710 had 3 target genes. To better explain the effect of RSV on the apoptosis of POGCs, we screened “organic substance metabolic process”-related miRNAs (Supplementary Table S7) and their target genes based on the key miRNAs enriched in the apoptosis signaling pathway and constructed a network (Figure 8B; Supplementary Table S7). In the “organic substance metabolic process” function, we found 118 and 83 target genes for ssc-miR-34a and ssc-miR-143-5p, respectively. In addition, ssc-miR-34a and ssc-miR-143-5p shared three common target genes: MLIT1, ZGPAT, and CLCN2.

## 4 Discussion

Granulosa cells are important in follicular development and atresia. Apoptosis of granulosa cells is closely associated with follicular atresia (Wang et al., 2023), process controlled by reproductive hormones and cytokines (Santos et al., 2022; Bevilaqua et al., 2023; Grynberg et al., 2023; Li et al., 2023). Numerous studies performed in chicken, rodents and pigs have shown that proapoptotic and antiapoptotic factors contribute to follicular atresia triggering the apoptosis of granulosa cells (Guo et al., 2019; Han et al., 2023; Ludwig et al., 2023). In the current study, we aimed, with the use of sRNA sequencing, to examine the effect of natural polyphenol RSV on apoptosis of porcine granulosa cells.

RSV effects have been extensively studied on cells and tissues, and RSV can not only act as a natural antioxidant at low concentrations in normal cells, but also act as a pro-oxidant to increase apoptosis (Li et al., 2022; Yuan et al., 2022; Zareifi et al., 2022). Studies have shown that RSV can promote cell apoptosis and inhibit cell proliferation *in vitro* (Wong et al., 2010; Liu et al., 2019; Muñoz-López et al., 2022), and RSV exerts its effects by interacting with multiple cellular targets and modulating various signal transduction pathways (Haworth and Avkiran, 2001). RSV and its derivatives can protect cells from mitochondrial ROS through SIRT1, and affect the metabolism of skeletal muscle, adipose tissue and liver (Song et al., 2018; Zhou et al., 2019; Hosoda et al., 2021). RSV also has the effects of regulating angiogenesis and anti-inflammation (Shi et al., 2022). There are also many studies reporting that exogenous RSV can regulate the expression of miRNA in different tissues (Karius et al., 2012; Lançon et al., 2013; Milenkovic et al., 2013; Altamemi et al., 2014; Latruffe et al., 2015). Based on numerous studies on RSV, some scholars pointed out that miRNAs may act as RSV targets in ovarian tissues and are involved in controlling signal transduction mechanisms (Chaichian et al., 2022).

In the current study, we identified differential expression profiles of miRNAs in POGCs treated with RSV. In addition, these profiles differed between the two examined RSV concentration. We found that the top 20 DE-miRNAs expressed in the LOW vs. CON group included miRNAs that have been reported in other species, such as miR-190a-5p, miR-503, miR-107, miR-17-5p, and ssc-miR-202-3p. Among them, miR-503 was localized to both granulosa cells and oocytes in mice (Lei et al., 2010), and high levels of miR-503 also led to the decrease of target gene expression, such Cdkn1b and Ccnd2. These showed that miR-503 plays a role in follicle granulosa cell proliferation and differentiation. MiR-107 is considered a key miRNA for the regulation of follicular selection (Li et al., 2019) and targets the expression of lipid regulation-related genes such as HSD17B12, ALDH5A1, and LIPC. MiR-202-3p is expressed in chicken ovarian follicles, and its target MMPs and ADAMs leading to the progression of follicle development, ovulation, or atresia. (Ocłoń and Hrabia, 2021). In addition, miR-17-5p has been reported to be differentially expressed in the preovulatory ovarian follicles of Large White and Chinese Taihu sows and has been confirmed to regulate POGC growth and estradiol synthesis through targeting the E2F1 gene in pig ovaries (Zhang S. et al., 2019). MiR-190a-5p was the most downregulated miRNA in the LOW vs. CON group, and in the eight-week-old male mice, RSV inhibited the upregulation of miR-190a-5p caused by TGF-β1. The decreased expression of miR-190a-5p could further enhance HGF expression and reduce hepatocyte apoptosis (Liang et al., 2022). This suggests that RSV may promote POGC apoptosis through downregulating the expression of miR-190a-5p. What’s more, the top 20 DE-miRNAs such as miR-378 and miR-142-5p were expressed in the HIGH vs. CON group, and have also been reported to be associated with ovarian development, cell proliferation, and apoptosis. It has been reported that miR-378 and its target gene CYP19A1 regulate steroidogenesis, cell survival, and differentiation during follicle selection and ovulation (Schauer et al., 2013). Previous studies have demonstrated an association between high miR-378 levels and reduced estradiol levels in subordinate follicles (Xu et al., 2011). Alternatively, increased miR-378 levels in subordinate follicles may be associated with a reported role in mediating cell death in some tissues (Knezevic et al., 2012). MiR-142-5p is expressed at higher levels in atretic follicles than in healthy follicles, its target IGF1 and PAPPA putatively involved in follicular atresia (Donadeu et al., 2017). MiR-378-3p is expressed in POGCs, but its expression decreases during maturation; meanwhile, targeting of the progesterone receptor by miR-378-3p decreased downstream gene expression (Toms et al., 2015). In our study, miR-142-5p and miR-378-3p were upregulated and downregulated, respectively, and may play a role in mediating follicular development and apoptosis of ovarian granulosa cells. Our study explored the function of DE-miRNAs in POGC apoptosis induced by RSV and these results offer novel insights into the involvement of miRNAs in the apoptosis of ovarian granulosa cells.

To further understand the potential function of the DE-miRNAs, their predicted genes were annotated by KEGG and GO. In the LOW vs. CON group, we found that DE-miRNAs’ target genes were involved in various functions related to cell development. Among them, miR-503 (Lei et al., 2010), miR-107 (Li et al., 2019), and miR-17-5p (Zhang S. et al., 2019) are closely related to ovarian development and granulosa cell apoptosis. In the HIGH vs. CON group, the enriched GO functions of upregulated and downregulated DE-miRNAs were related to responses to stimuli and metabolic processes. When POGCs were co-cultured with high concentrations of RSV, the POGCs responded to the organic stimulus and the metabolic process was changed. However, the upregulated DE-miRNA target genes enriched in GO terms were related to the regulation of protein binding, cell communication, cellular metabolic processes, and signal transduction, indicating that a large number of genes with key roles in POGCs apoptosis induced by RSV are highly expressed in POGCs. These results suggest that POGCs apoptosis induced by RSV may be regulated by these miRNAs.

KEGG analysis showed that DE-miRNAs’ target genes were involved in signaling pathways related to ovarian granulosa cell functions and hormonal regulation such as the mTOR signaling pathway, PI3K-Akt signaling pathway, MAPK signaling pathway, AMPK signaling pathway, Wnt signaling pathway, TNF signaling pathway, and apoptosis signaling pathway. For instance, the Wnt signaling pathway is enriched by upregulated DE-miRNAs and includes 45 genes that are known to be involved in mammalian reproduction, including follicular development and ovulation (Kobayashi et al., 2011). Similarly, Wnt-4 regulates the function of ovarian granulosa cells at specific stages of follicular development in the rodent ovary (Hsieh et al., 2002). Interestingly, the apoptosis and PI3K-Akt signaling pathways were enriched by the target genes of DE-miRNAs in the LOW or HIGH vs. CON groups. These signaling pathways play key roles in the proliferation and apoptosis of mammalian cells during follicular growth and in the promotion of primordial follicle activation. These data clarify the potential roles of DE-miRNAs in POGCs apoptosis. In ovaries, many genes and associated signaling pathways are involved in the growth of ovarian follicles until ovulation. Therefore, the molecular functions of the predicted targets and pathways of miRNAs require further validation.

As a flavonoid polyphenol, RSV may be related to the potential mechanism of POGCs apoptosis induced by metabolism of organic substances. And we focused on apoptosis signaling pathway and RSV-related organic substance metabolic process to find key miRNAs involved in RSV-induced apoptosis in POGCs. Then, we constructed miRNA-mRNA networks for “Apoptosis” and “Metabolism”. In the Apoptosis-related network, we found 17 miRNAs involved in the pathway targeting 27 genes, and ssc-miR-34a, ssc-miR-1285, ssc-miR-143-5p, ssc-miR-320, and novel_710 were selected. Among them, ssc-miR-34a and ssc-miR-143-5p, with the most target genes in the HIGH vs. CON group, were selected as key miRNAs, and an RSV-related metabolic network was constructed. We identified three genes that shared three target genes: MLIT1, ZGPAT, and CLCN2. These results verified that RSV is extensively involved in the regulation of miRNA and gene expression during apoptosis in POGCs.

In brief, small RNA sequencing results suggest that DE-miRNAs may play a crucial role in the regulation of RSV-induced apoptosis in POGCs by regulating their target genes to participate in metabolism-related and apoptosis-related signaling pathways. Nevertheless, since the results of the present study are based on sRNA-seq data and their analysis, additional functional studies (e.g., feeding experiments, miRNA overexpression or interference) are required to confirm the potential involvement of particular miRNAs in the regulation of follicular functions in pigs. The presented results suggest that RSV is important in the regulation of reproductive processes in pigs.

## 5 Conclusion

Overall, this study provided an improved understanding of the molecular mechanisms underlying the impact of RSV on miRNA expression to regulate POGCs apoptosis. We constructed the key miRNA-mRNA networks involved in apoptosis and metabolism, furthermore, ssc-miR-34a and ssc-miR-143-5p, which had the highest number of target genes, were selected as key miRNAs. These results provide a better understanding of the roles of miRNAs and RSV in ovarian granulosa cell development in pigs.

## Data Availability

The datasets presented in this study can be found in online repositories. The names of the repository/repositories and accession number(s) can be found below: BioProject accession number: PRJNA813031.

## References

[B1] Aires V. Delmas D. Djouadi F. Bastin J. Cherkaoui-Malki M. Latruffe N. (2017). Resveratrol-induced changes in MicroRNA expression in primary human fibroblasts harboring carnitine-palmitoyl transferase-2 gene mutation, leading to fatty acid oxidation deficiency. Molecules 23 (1), 7. 10.3390/molecules23010007 29271911 PMC5943968

[B2] Altamemi I. Murphy E. A. Catroppo J. F. Zumbrun E. E. Zhang J. McClellan J. L. (2014). Role of microRNAs in resveratrol-mediated mitigation of colitis-associated tumorigenesis in Apc(Min/+) mice. J. Pharmacol. Exp. Ther. 350 (1), 99–109. 10.1124/jpet.114.213306 24817032 PMC4056272

[B3] Bai Y. Pan B. Zhan X. Silver H. Li J. (2021). MicroRNA 195-5p targets Foxo3 promoter region to regulate its expression in granulosa cells. Int. J. Mol. Sci. 22 (13), 6721. 10.3390/ijms22136721 34201585 PMC8267755

[B4] Bartel D. P. (2004). MicroRNAs: Genomics, biogenesis, mechanism, and function. Cell 116 (2), 281–297. 10.1016/s0092-8674(04)00045-5 14744438

[B5] Basini G. Tringali C. Baioni L. Bussolati S. Spatafora C. Grasselli F. (2010). Biological effects on granulosa cells of hydroxylated and methylated resveratrol analogues. Mol. Nutr. Food Res. 54, S236–S243. 10.1002/mnfr.200900320 20140899

[B6] Bevilaqua J. R. Rodriguez M. G. K. Maciel G. S. Vergani G. B. Fonseca J. F. D. Bartlewski P. M. (2023). Luteal function, biometrics, and echotextural attributes in santa inês ewes superovulated with different total doses of porcine follicle-stimulating hormone. Anim. (Basel) 13 (5), 873. 10.3390/ani13050873 PMC10000133 36899731

[B7] Brennecke J. Hipfner D. R. Stark A. Russell R. B. Cohen S. M. (2003). Bantam encodes a developmentally regulated microRNA that controls cell proliferation and regulates the proapoptotic gene hid in Drosophila. Cell 113 (1), 25–36. 10.1016/s0092-8674(03)00231-9 12679032

[B8] Chaichian S. Bidgoli S. A. Nikfar B. Moazzami B. (2022). Lncrnas and mirnas: New targets for resveratrol in ovarian cancer research. Curr. Med. Chem. 30, 3238–3248. 10.2174/1389201024666221111160407 36372916

[B9] Chen C. Z. Li L. Lodish H. F. Bartel D. P. (2004). MicroRNAs modulate hematopoietic lineage differentiation. Science 303 (5654), 83–86. 10.1126/science.1091903 14657504

[B10] Donadeu F. X. Mohammed B. T. Ioannidis J. (2017). A miRNA target network putatively involved in follicular atresia. Domest. Anim. Endocrinol. 58, 76–83. 10.1016/j.domaniend.2016.08.002 27664382 PMC5145806

[B11] Fabová Z. Loncová B. Bauer M. Sirotkin A. V. (2023). Involvement of microRNA miR-125b in the control of porcine ovarian cell functions. Gen. Comp. Endocrinol. 334, 114215. 10.1016/j.ygcen.2023.114215 36669691

[B12] Gatouillat G. Balasse E. Joseph-Pietras D. Morjani H. Madoulet C. (2010). Resveratrol induces cell-cycle disruption and apoptosis in chemoresistant B16 melanoma. J. Cell Biochem. 110 (4), 893–902. 10.1002/jcb.22601 20564188

[B13] Grynberg M. Pytel S. Peigne M. Sonigo C. (2023). The follicular output rate in normo-ovulating women undergoing ovarian stimulation is increased after unilateral oophorectomy. Hum. Reprod., dead056. 10.1093/humrep/dead056 36961937

[B14] Guo T. Zhang J. Yao W. Du X. Li Q. Huang L. (2019). CircINHA resists granulosa cell apoptosis by upregulating CTGF as a ceRNA of miR-10a-5p in pig ovarian follicles. Biochim. Biophys. Acta Gene Regul. Mech. 1862 (10), 194420. 10.1016/j.bbagrm.2019.194420 31476383

[B15] Gusman J. Malonne H. Atassi G. (2001). A reappraisal of the potential chemopreventive and chemotherapeutic properties of resveratrol. Carcinogenesis 22 (8), 1111–1117. 10.1093/carcin/22.8.1111 11470738

[B16] Han S. Zhao X. Zhang Y. Amevor F. K. Tan B. Ma M. (2023). MiR-34a-5p promotes autophagy and apoptosis of ovarian granulosa cells via the Hippo-YAP signaling pathway by targeting LEF1 in chicken. Poult. Sci. 102 (2), 102374. 10.1016/j.psj.2022.102374 36529101 PMC9791594

[B17] Haworth R. S. Avkiran M. (2001). Inhibition of protein kinase D by resveratrol. Biochem. Pharmacol. 62 (12), 1647–1651. 10.1016/s0006-2952(01)00807-3 11755118

[B18] Hosoda R. Hamada H. Uesugi D. Iwahara N. Nojima I. Horio Y. (2021). Different antioxidative and antiapoptotic effects of piceatannol and resveratrol. J. Pharmacol. Exp. Ther. 376 (3), 385–396. 10.1124/jpet.120.000096 33335015

[B19] Hsieh M. Johnson M. A. Greenberg N. M. Richards J. S. (2002). Regulated expression of Wnts and Frizzleds at specific stages of follicular development in the rodent ovary. Endocrinology 143 (3), 898–908. 10.1210/endo.143.3.8684 11861511

[B63] Hu H. Fu Y. Zhou B. Li Z. Liu Z. Jia Q. (2021). Long non-coding RNA TCONS_00814106 regulates porcine granulosa cell proliferation and apoptosis by sponging miR-1343. Mol. Cell Endocrinol. 520, 111064. 10.1016/j.mce.2020.111064 33091558

[B20] Jin X. Wang K. Liu H. Hu F. Zhao F. Liu J. (2016). Protection of bovine mammary epithelial cells from hydrogen peroxide-induced oxidative cell damage by resveratrol. Oxid. Med. Cell Longev. 2016, 2572175. 10.1155/2016/2572175 26962394 PMC4707352

[B21] Joe A. K. Liu H. Suzui M. Vural M. E. Xiao D. Weinstein I. B. (2002). Resveratrol induces growth inhibition, S-phase arrest, apoptosis, and changes in biomarker expression in several human cancer cell lines. Clin. Cancer Res. 8 (3), 893–903. 11895924

[B22] Karius T. Schnekenburger M. Dicato M. Diederich M. (2012). MicroRNAs in cancer management and their modulation by dietary agents. Biochem. Pharmacol. 83 (12), 1591–1601. 10.1016/j.bcp.2012.02.004 22342289

[B23] Knezevic I. Patel A. Sundaresan N. R. Gupta M. P. Solaro R. J. Nagalingam R. S. (2012). A novel cardiomyocyte-enriched microRNA, miR-378, targets insulin-like growth factor 1 receptor: Implications in postnatal cardiac remodeling and cell survival. J. Biol. Chem. 287 (16), 12913–12926. 10.1074/jbc.M111.331751 22367207 PMC3339988

[B24] Kobayashi A. Stewart C. A. Wang Y. Fujioka K. Thomas N. C. Jamin S. P. (2011). β-Catenin is essential for Müllerian duct regression during male sexual differentiation. Development 138 (10), 1967–1975. 10.1242/dev.056143 21490063 PMC3082302

[B25] Kong X. X. Fu Y. C. Xu J. J. Zhuang X. L. Chen Z. G. Luo L. L. (2011). Resveratrol, an effective regulator of ovarian development and oocyte apoptosis. J. Endocrinol. Invest. 34 (11), e374–e381. 10.3275/7853 21738004

[B26] Lançon A. Michaille J. J. Latruffe N. (2013). Effects of dietary phytophenols on the expression of microRNAs involved in mammalian cell homeostasis. J. Sci. Food Agric. 93 (13), 3155–3164. 10.1002/jsfa.6228 23674481

[B27] Langmead B. Trapnell C. Pop M. Salzberg S. L. (2009). Ultrafast and memory-efficient alignment of short DNA sequences to the human genome. Genome Biol. 10 (3), R25. 10.1186/gb-2009-10-3-r25 19261174 PMC2690996

[B28] Larrosa M. Tomas-Barberan F. A. Espin J. C. (2003). Grape polyphenol resveratrol and the related molecule 4-hydroxystilbene induce growth inhibition, apoptosis, S-phase arrest, and upregulation of cyclins A, E, and B1 in human SK-Mel-28 melanoma cells. J. Agric. Food Chem. 51 (16), 4576–4584. 10.1021/jf030073c 14705880

[B29] Latruffe N. Lançon A. Frazzi R. Aires V. Delmas D. Michaille J. J. (2015). Exploring new ways of regulation by resveratrol involving miRNAs, with emphasis on inflammation. Ann. N. Y. Acad. Sci. 1348 (1), 97–106. 10.1111/nyas.12819 26190093

[B30] Lee E. J. Min H. Y. Joo Park H. Chung H. J. Kim S. Nam Han Y. (2004). G2/M cell cycle arrest and induction of apoptosis by a stilbenoid, 3,4,5-trimethoxy-4'-bromo-cis-stilbene, in human lung cancer cells. Life Sci. 75 (23), 2829–2839. 10.1016/j.lfs.2004.07.002 15464834

[B31] Lei L. Jin S. Gonzalez G. Behringer R. R. Woodruff T. K. (2010). The regulatory role of Dicer in folliculogenesis in mice. Mol. Cell Endocrinol. 315 (1-2), 63–73. 10.1016/j.mce.2009.09.021 19799966 PMC2814883

[B32] Li Q. Hu S. Wang Y. Deng Y. Yang S. Hu J. (2019). mRNA and miRNA transcriptome profiling of granulosa and theca layers from geese ovarian follicles reveals the crucial pathways and interaction networks for regulation of follicle selection. Front. Genet. 10, 988. 10.3389/fgene.2019.00988 31708963 PMC6820619

[B33] Li Z. Dong J. Wang M. Yan J. Hu Y. Liu Y. (2022). Resveratrol ameliorates liver fibrosis induced by nonpathogenic Staphylococcus in BALB/c mice through inhibiting its growth. Mol. Med. 28 (1), 52. 10.1186/s10020-022-00463-y 35508992 PMC9066969

[B34] Li X. Zhu Y. Zhao T. Zhang X. Qian H. Wang J. (2023). Role of COX-2/PGE2 signaling pathway in the apoptosis of rat ovarian granulosa cells induced by MEHP. Ecotoxicol. Environ. Saf. 254, 114717. 10.1016/j.ecoenv.2023.114717 36889213

[B35] Liang F. Xu X. Tu Y. (2022). Resveratrol inhibited hepatocyte apoptosis and alleviated liver fibrosis through miR-190a-5p/HGF axis. Bioorg Med. Chem. 57, 116593. 10.1016/j.bmc.2021.116593 35093804

[B36] Liu J. Yao W. Yao Y. Du X. Zhou J. Ma B. (2014). MiR-92a inhibits porcine ovarian granulosa cell apoptosis by targeting Smad7 gene. FEBS Lett. 588 (23), 4497–4503. 10.1016/j.febslet.2014.10.021 25448599

[B37] Liu X. Li H. Wu M. L. Wu J. Sun Y. Zhang K. L. (2019). Resveratrol reverses retinoic acid resistance of anaplastic thyroid cancer cells via demethylating CRABP2 gene. Front. Endocrinol. (Lausanne) 10, 734. 10.3389/fendo.2019.00734 31736873 PMC6828648

[B38] Ludwig C. L. M. Bohleber S. Lapp R. Rebl A. Wirth E. K. Langhammer M. (2023). Alterations in gonadotropin, apoptotic and metabolic pathways in granulosa cells warrant superior fertility of the Dummerstorf high fertility mouse line 1. J. Ovarian Res. 16 (1), 32. 10.1186/s13048-023-01113-5 36739419 PMC9898973

[B39] Manna S. K. Mukhopadhyay A. Aggarwal B. B. (2000). Resveratrol suppresses TNF-induced activation of nuclear transcription factors NF-kappa B, activator protein-1, and apoptosis: Potential role of reactive oxygen intermediates and lipid peroxidation. J. Immunol. 164 (12), 6509–6519. 10.4049/jimmunol.164.12.6509 10843709

[B40] Milenkovic D. Jude B. Morand C. (2013). miRNA as molecular target of polyphenols underlying their biological effects. Free Radic. Biol. Med. 64, 40–51. 10.1016/j.freeradbiomed.2013.05.046 23751562

[B41] Muñoz-López S. Sánchez-Melgar A. Martín M. Albasanz J. L. (2022). Resveratrol enhances A(1) and hinders A(2A) adenosine receptors signaling in both HeLa and SH-SY5Y cells: Potential mechanism of its antitumoral action. Front. Endocrinol. (Lausanne) 13, 1007801. 10.3389/fendo.2022.1007801 36407311 PMC9669387

[B42] Ocłoń E. Hrabia A. (2021). miRNA expression profile in chicken ovarian follicles throughout development and miRNA-mediated MMP expression. Theriogenology 160, 116–127. 10.1016/j.theriogenology.2020.11.004 33217625

[B43] Poy M. N. Eliasson L. Krutzfeldt J. Kuwajima S. Ma X. Macdonald P. E. (2004). A pancreatic islet-specific microRNA regulates insulin secretion. Nature 432 (7014), 226–230. 10.1038/nature03076 15538371

[B44] Santos P. H. Nunes S. G. Franchi F. F. Giroto A. B. Fontes P. K. Pinheiro V. G. (2022). Expression of bta-miR-222 and LHCGR in bovine cultured granulosa cells: Impact of follicle deviation and regulation by FSH/insulin *in vitro* . Theriogenology 182, 71–77. 10.1016/j.theriogenology.2022.01.034 35131675

[B45] Sarkar C. Pal S. (2014). Ameliorative effect of resveratrol against fluoride-induced alteration of thyroid function in male wistar rats. Biol. Trace Elem. Res. 162 (1-3), 278–287. 10.1007/s12011-014-0108-3 25164033

[B46] Schauer S. N. Sontakke S. D. Watson E. D. Esteves C. L. Donadeu F. X. (2013). Involvement of miRNAs in equine follicle development. Reproduction 146 (3), 273–282. 10.1530/rep-13-0107 23813447

[B47] Shi J. Wang J. Jia N. Sun Q. (2022). A network pharmacology study on mechanism of resveratrol in treating preeclampsia via regulation of AGE-RAGE and HIF-1 signalling pathways. Front. Endocrinol. (Lausanne) 13, 1044775. 10.3389/fendo.2022.1044775 36686428 PMC9849370

[B48] Singh S. K. Banerjee S. Acosta E. P. Lillard J. W. Singh R. (2017). Resveratrol induces cell cycle arrest and apoptosis with docetaxel in prostate cancer cells via a p53/p21WAF1/CIP1 and p27KIP1 pathway. Oncotarget 8 (10), 17216–17228. 10.18632/oncotarget.15303 28212547 PMC5370034

[B49] Sirotkin A. V. (2021). Effects of resveratrol on female reproduction: A review. Phytother. Res. 35 (10), 5502–5513. 10.1002/ptr.7185 34101259

[B50] Song J. Yang B. Jia X. Li M. Tan W. Ma S. (2018). Distinctive roles of sirtuins on diabetes, protective or detrimental? Front. Endocrinol. (Lausanne) 9, 724. 10.3389/fendo.2018.00724 30559718 PMC6284472

[B51] Toms D. Xu S. Pan B. Wu D. Li J. (2015). Progesterone receptor expression in granulosa cells is suppressed by microRNA-378-3p. Mol. Cell Endocrinol. 399, 95–102. 10.1016/j.mce.2014.07.022 25150622

[B52] Wang C. Sun H. Davis J. S. Wang X. Huo L. Sun N. (2023). FHL2 deficiency impairs follicular development and fertility by attenuating EGF/EGFR/YAP signaling in ovarian granulosa cells. Cell Death Dis. 14 (4), 239. 10.1038/s41419-023-05759-3 37015904 PMC10073124

[B53] Wong D. H. Villanueva J. A. Cress A. B. Duleba A. J. (2010). Effects of resveratrol on proliferation and apoptosis in rat ovarian theca-interstitial cells. Mol. Hum. Reprod. 16 (4), 251–259. 10.1093/molehr/gaq002 20067985 PMC2834407

[B54] Xu S. Linher-Melville K. Yang B. B. Wu D. Li J. (2011). Micro-RNA378 (miR-378) regulates ovarian estradiol production by targeting aromatase. Endocrinology 152 (10), 3941–3951. 10.1210/en.2011-1147 21846797 PMC3176644

[B55] Yuan W. Zhang M. Wang C. Li B. Li L. Ye F. (2022). Resveratrol attenuates high-fat diet-induced hepatic lipotoxicity by upregulating bmi-1 expression. J. Pharmacol. Exp. Ther. 381 (2), 96–105. 10.1124/jpet.121.001018 35221291

[B56] Zareifi D. S. Chaliotis O. Chala N. Meimetis N. Sofotasiou M. Zeakis K. (2022). A network-based computational and experimental framework for repurposing compounds toward the treatment of non-alcoholic fatty liver disease. iScience 25 (3), 103890. 10.1016/j.isci.2022.103890 35252807 PMC8889147

[B57] Zhang J. Xu Y. Liu H. Pan Z. (2019a). MicroRNAs in ovarian follicular atresia and granulosa cell apoptosis. Reprod. Biol. Endocrinol. 17 (1), 9. 10.1186/s12958-018-0450-y 30630485 PMC6329178

[B58] Zhang S. Wang L. Wang L. Chen Y. Li F. (2019b). miR-17-5p affects porcine granulosa cell growth and oestradiol synthesis by targeting E2F1 gene. Reprod. Domest. Anim. 54 (11), 1459–1469. 10.1111/rda.13551 31424586

[B59] Zhang J. Q. Gao B. W. Guo H. X. Ren Q. L. Wang X. W. Chen J. F. (2020). miR-181a promotes porcine granulosa cell apoptosis by targeting TGFBR1 via the activin signaling pathway. Mol. Cell Endocrinol. 499, 110603. 10.1016/j.mce.2019.110603 31574295

[B60] Zhang H. Liu Y. Han Z. Xu Q. Zhang N. Wang J. (2023). Integrated analysis of lncRNA and mRNA for the apoptosis of porcine ovarian granulosa cells after polyphenol resveratrol treatment. Front. Vet. Sci. 9, 1065001. 10.3389/fvets.2022.1065001 36704707 PMC9872129

[B61] Zhou L. Xiao X. Zhang Q. Zheng J. Deng M. (2019). Deciphering the anti-obesity benefits of resveratrol: The "gut microbiota-adipose tissue" Axis. Front. Endocrinol. (Lausanne) 10, 413. 10.3389/fendo.2019.00413 31316465 PMC6610334

[B62] Zhu W. Yang M. Shang J. Xu Y. Wang Y. Tao Q. (2019). MiR-222 inhibits apoptosis in porcine follicular granulosa cells by targeting the THBS1 gene. Anim. Sci. J. 90 (6), 719–727. 10.1111/asj.13208 30983045

