# Supplementary material for: Identification of microRNAs implicated in modulating resveratrol-induced apoptosis in porcine granulosa cells
Source: Front Cell Dev Biol. 2023 May 5;11:1169745. doi: 10.3389/fcell.2023.1169745 (PMC10211428; doi:10.3389/fcell.2023.1169745)

Supplementary Material

Identification of microRNAs Implicated in Modulating Resveratrol-Induced Apoptosis in Porcine Granulosa Cells

Huibin Zhang^1,2,†^, Jinglin Wang^1,2,†^, Fan Xie^1,2^, Yangguang Liu^1,2^, Mengyao Qiu^1,2^, Zheng Han^1,2^, Yueyun Ding1,2, Xianrui Zheng^1,2^, Zongjun Yin^1,2*^, Xiaodong Zhang^1,2*^

*** Correspondence:**Zongjun Yin, yinzongjun@ahau.edu.cn
Xiaodong Zhang, xdzhang1983@163.com

# 1 Supplementary Tables

**Table S1**. The overview of small RNA-seq data.

**Table S2**. All identified miRNAs and their expression levels

**Table S3**. All identified miRNAs. (A) All identified miRNAs in LOW vs. CON. (B) All identified miRNAs in HIGH vs. CON.

**Table S4**. Lists of DE-miRNAs’ target genes. (A) Lists of up-regulated DE-miRNAs’ target genes in LOW vs. CON. (B) Lists of down-regulated DE-miRNAs’ target genes in LOW vs. CON. (C) Lists of up-regulated DE-miRNAs’ target genes in HIGH vs. CON. (D) Lists of down-regulated DE-miRNAs’ target genes in HIGH vs. CON.

**Table S5**. The GO and KEGG analyses of DE-miRNAs in LOW vs. CON. (A) Up-regulated DE-miRNAs' GO. (B) Down -regulated DE-miRNAs' GO. (C) Up-regulated DE-miRNAs' KEGG. (D) Down-regulated DE-miRNAs' KEGG.

**Table S6**. The GO and KEGG analyses of DE-miRNAs in HIGH vs. CON. (A) Up-regulated DE-miRNAs' GO. (B) Down -regulated DE-miRNAs' GO. (C) Up-regulated DE-miRNAs' KEGG. (D) Down-regulated DE-miRNAs' KEGG.

**Table S7**. miRNA-mRNA network for apoptosis. (A) Apoptosis network. (B) Metabolic network.

**Table S8**. The primers of reference gene and miRNAs

# 2 Supplementary Figures

**Supplementary Figure 1. TPM density distribution of miRNA**


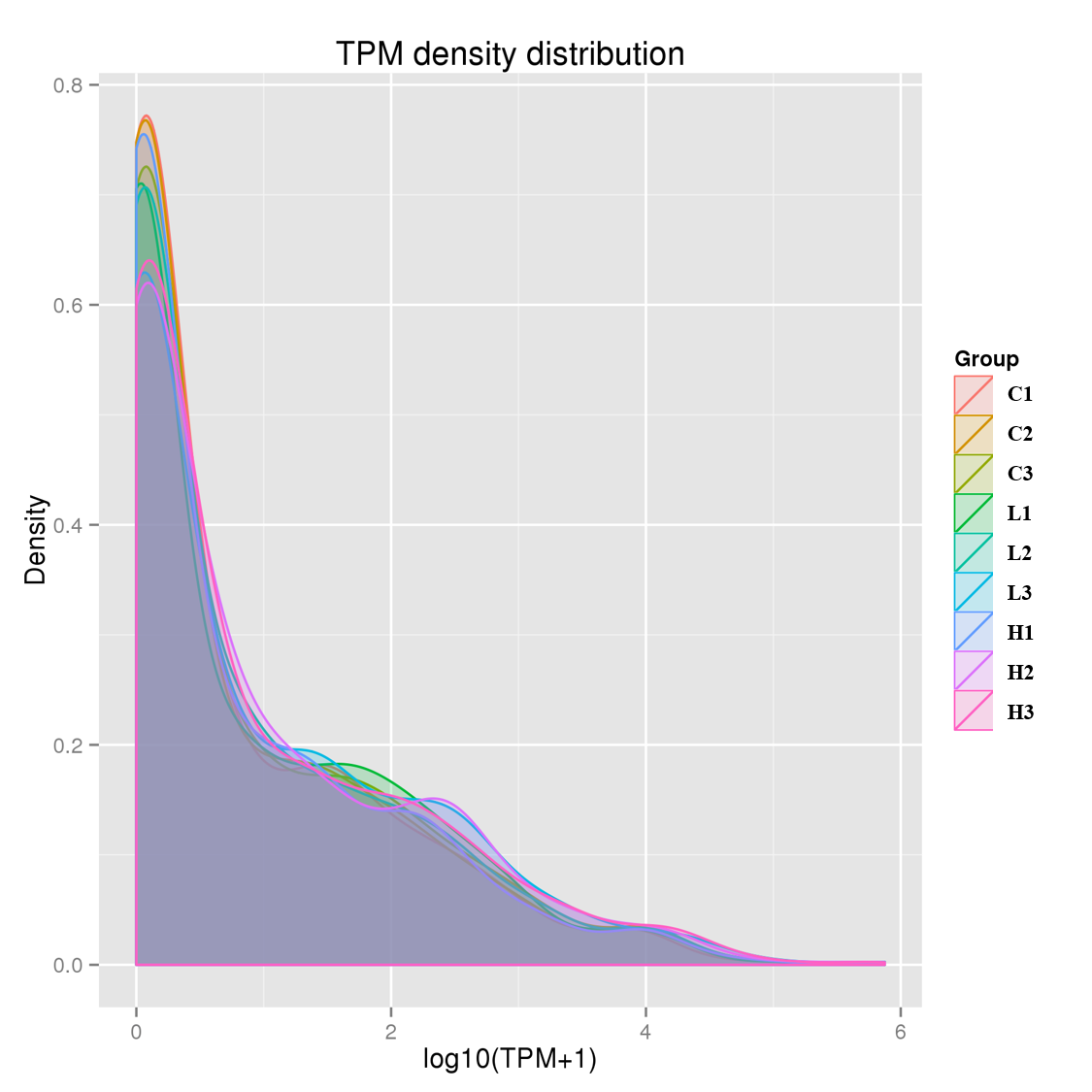


**Supplementary Figure 2. Cluster heatmap of differentially expressed sRNA**


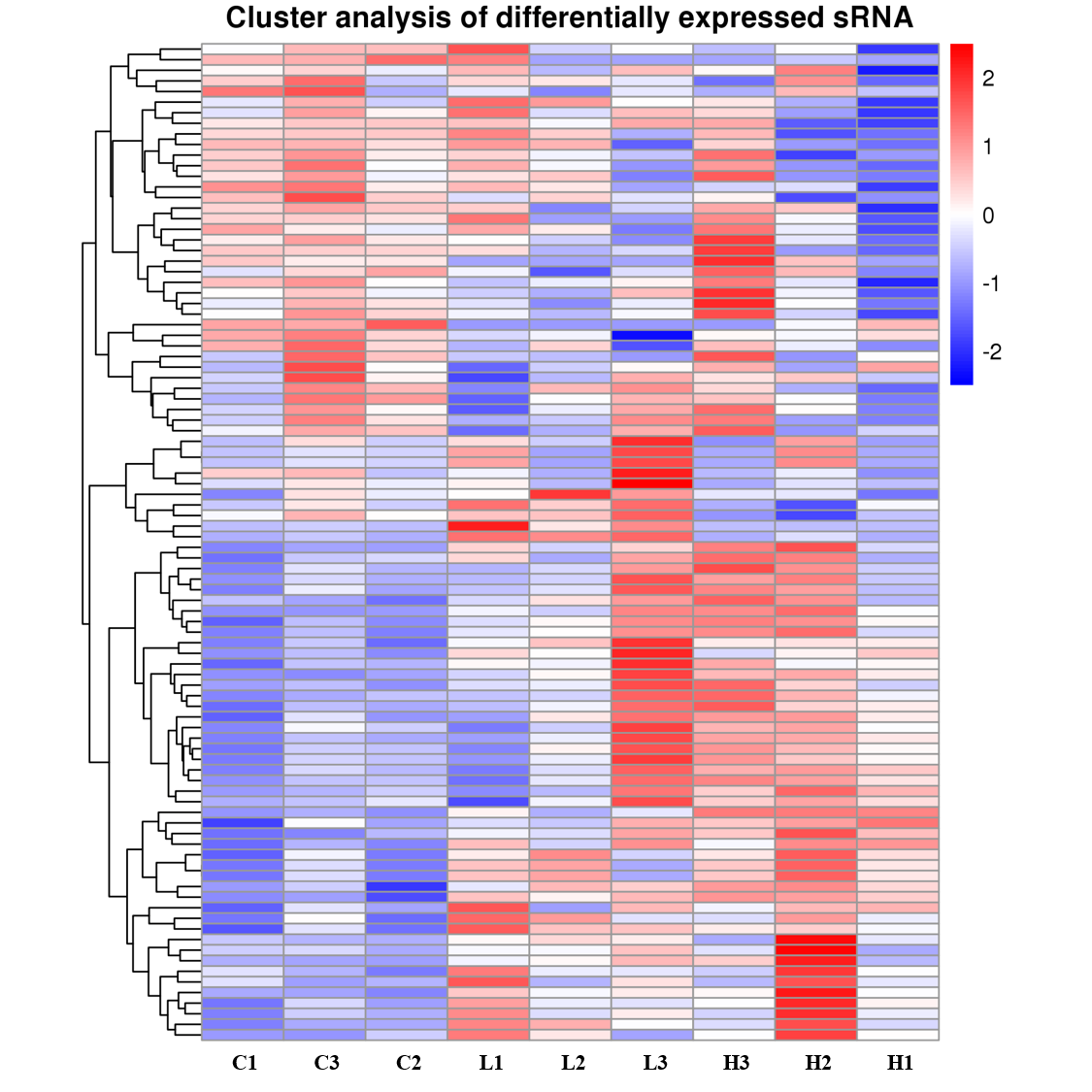

Supplement: Supplementary file 9 [file DataSheet1.docx]
